# The South China Sea is not a mini-Atlantic: plate-edge rifting *vs* intra-plate rifting

**DOI:** 10.1093/nsr/nwz135

**Published:** 2019-09-12

**Authors:** Pinxian Wang, Chi-Yue Huang, Jian Lin, Zhimin Jian, Zhen Sun, Minghui Zhao

**Affiliations:** 1 State Key Laboratory of Marine Geology, Tongji University, Shanghai 200092, China; 2 Department of Earth Sciences, Cheng Kung University, Tainan 701, China; 3 Department of Geology and Geophysics, Woods Hole Oceanographic Institution, Woods Hole, MA 02543-1050, USA; 4 Key Laboratory of Ocean and Marginal Sea Geology, South China Sea Institute of Oceanology, Chinese Academy of Sciences, Guangzhou 510301, China

**Keywords:** rifting, marginal basin, passive margin, South China Sea, Western Pacific, subduction

## Abstract

The South China Sea, as ‘a non-volcanic passive margin basin’ in the Pacific, has often been considered as a small-scale analogue of the Atlantic. The recent ocean drilling in the northern South China Sea margin found, however, that the Iberian model of non-volcanic rifted margin from the Atlantic does not apply to the South China Sea. In this paper, we review a variety of rifted basins and propose to discriminate two types of rifting basins: plate-edge type such as the South China Sea and intra-plate type like the Atlantic. They not only differ from each other in structure, formation process, lifespan and geographic size, but also occur at different stages of the Wilson cycle. The intra-plate rifting occurred in the Mesozoic and gave rise to large oceans, whereas the plate-edge rifting took place mainly in the mid-Cenozoic, with three-quarters of the basins concentrated in the Western Pacific. As a member of the Western Pacific system of marginal seas, the South China Sea should be studied not in isolation on its origin and evolution, but in a systematic context to include also its neighboring counterparts.

## INTRODUCTION

Continent break-up and basin formation are two of the fundamental processes in Earth tectonics. To understand the processes, the nature and structure of the basement are key elements but, in the deep ocean, the basement has been scientifically drilled only in very few basins due to technical limitation and major expenses; and thus the tectonic model is often based only on images from seismic reflection. Now the South China Sea (SCS) offers a unique opportunity. The International Ocean Discovery Programs (IODP) implemented three and a half drilling expeditions (IODP 349, 367, 368, 368X) there over the past 5 years to explore the processes of its formation. The acoustic basement was penetrated at 8 of the 12 drilled sites in its ocean basin or at the continent–ocean transition (COT), all in water depths exceeding 3700 m. This is the second large-scale drilling campaign of the world’s passive margin after several Ocean Drilling Program (ODP) legs to the North Atlantic in the 1990s.

The endeavor in the SCS has proved to be extremely rewarding. Beyond expectation, the drill results disproved the original assumption in the drilling proposal and inspired a new approach to the SCS tectonic research. Specifically, new IODP findings challenge the prevailing wisdom in applying the Atlantic model of basin opening to the SCS and call for a reconsideration of the process of its formation. Based on literature survey and the recent IODP results, this paper demonstrates the differences between the two types of ocean-basin formation, i.e. intra-plate *vs* plate-edge rifting, which are characteristic of two distinct stages in the Wilson cycle, respectively. We start with the drilling results in the SCS and show the differences between the SCS and the Atlantic. Then we trace back the research history of the SCS to identify the specific features of plate-edge rifting typical of marginal basins. Finally, we demonstrate that the Western Pacific marginal basins are interconnected as a system in their origin and evolution, and the processes of their formation can only be properly understood from inside this collective system.

**Figure 1:**
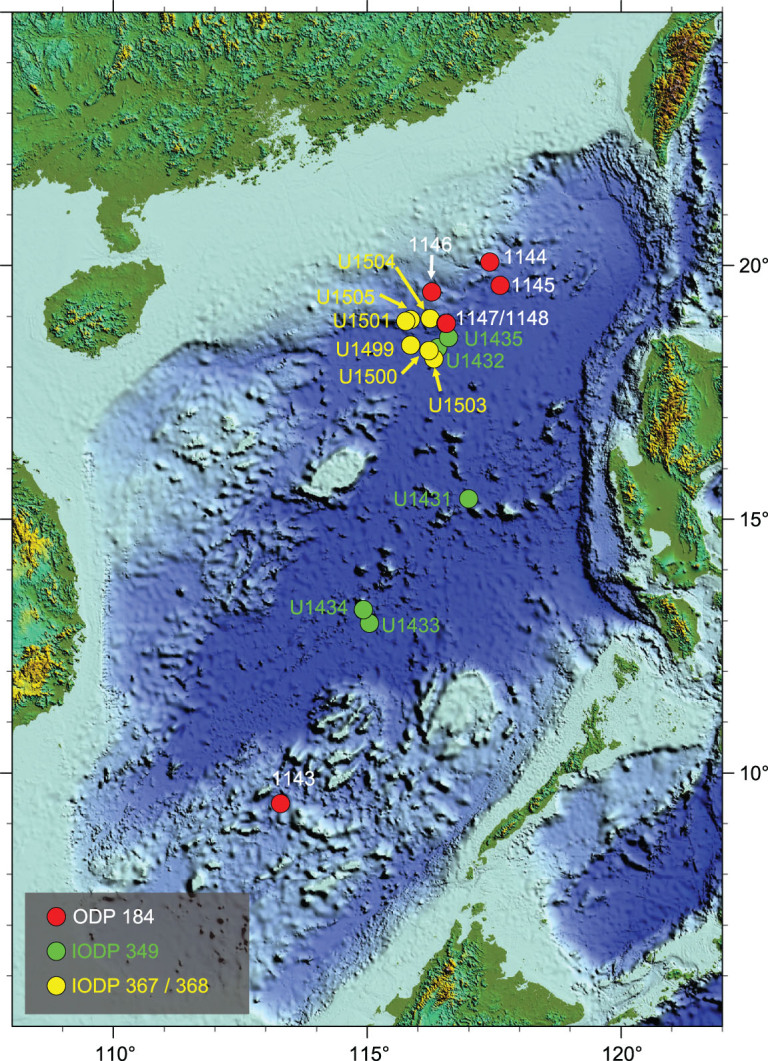
Scientific ocean-drilling sites in the South China Sea: ODP 184 in 1999, East Asian monsoon history; IODP 349 in 2014, SCS tectonics; IODP 367/368 in 2017, IODP 368X in 2018, SCS rifted margin.

## NEW FINDINGS OF OCEAN DRILLING

The SCS is certainly among the best-studied marginal basins in terms of offshore drilling. Aside from more than 2000 industrial drilling wells in its shelves and slopes, the deep basin has become the subject of scientific drilling since 1999. Over the last 20 years, a total of 17 sites were drilled and nearly 10 000 m of cores recovered, including 320 m of basement basalt (Fig. 1). Of particular interest are the recent IODP Expeditions 367, 368 and 368X to the northern continental margin, addressing questions relating to the rifting process and the rift-to-drift transition. The primary goal of these expeditions was ‘Testing hypotheses for lithosphere thinning during continental breakup’, namely to test the applicability of the Iberian model of non-volcanic passive margin to the SCS [1]. Contrary to expectations, however, the final results of drilling do not support this model.

The break-up of continental lithosphere and the opening of ocean basins have always been high priorities in ocean drilling and multi-decadal research in the Atlantic Ocean has yielded basic knowledge of basin formation in the passive margins. Two end-members have been recognized: volcanic or magma-rich and non-volcanic or magma-poor rifted margins. Volcanic rifted margins can be easily recognized by the seaward-dipping reflector (SDR) sequences in seismic transects; classical examples are the conjugate margins of East Greenland and northwestern Europe, where the break-up of continental lithosphere is linked to the Iceland mantle plume [2]. Less clear is the opening mechanism of the non-volcanic type, for which an alternative force is required to break up the lithosphere.

An important breakthrough was the development of the Iberian model of a magma-poor rifted margin, largely resulting from ocean drilling in the mid-1980s to 1990s. The model was developed on the basis of at least 16 drill sites of four DSDP (Deep Sea Drilling Program) and ODP legs at the Iberian margin of the North Atlantic spanning over 21 years, supported by extensive geophysical work. The Iberian model was applied to various parts of the world’s margins and has become the paradigm of the non-volcanic type of passive margin.

**Figure 2:**
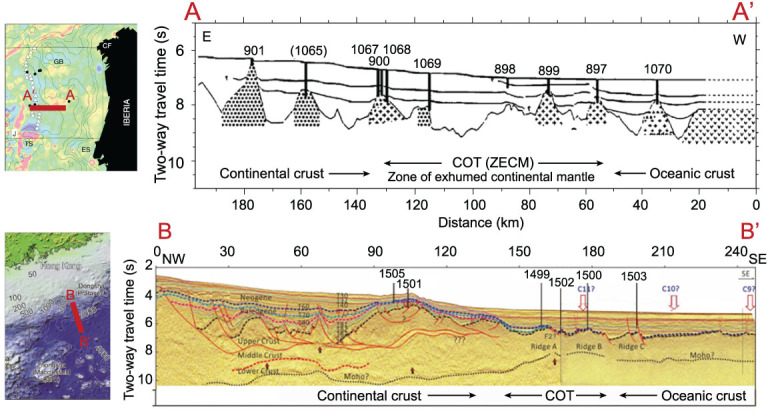
Comparison of interpreted seismic profiles crossing the drilling sites. (A-A’) Iberian margin, North Atlantic (based on [5]); (B-B′) northern margin of the SCS (based on [6,7,8]).

The Iberian model starts from a hypothetical ‘hyper extension’ of the continental lithosphere that dramatically thins the crust prior to break-up, together with the development of crust-cutting faults that allow water to penetrate into the subcontinental lithospheric mantle. Subsequent serpentinization and exhumation of mantle peridotite are hypothesized to lead to mechanical weakening of the mantle and the final rupture of the continental lithosphere [3,4]. This can be simply demonstrated along a transect of drill sites across the COT off the west Iberian margin (Fig. 2A-A’). Drilling at multiple sites in the wide COT has recovered serpentinized peridotite beneath post-rift sedimentary rocks; this is the key geological indicator of exhumed continental mantle hypothesized to be responsible for non-volcanic lithosphere break-up.

**Figure 3:**
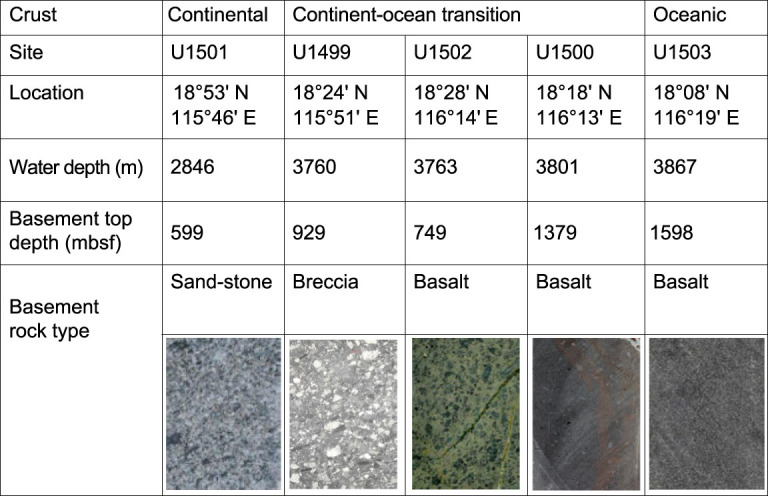
Basement rocks recovered at drill sites of IODP 367/368 (based on [6,7,8]).

Since the 1990s, the SCS has been considered as an ‘Atlantic-type’ passive margin basin. In recent years, the Iberian model has been invoked to interpret the rifting and rupturing of the SCS basin. Following the Iberian model, ‘mantle exhumation’ was speculated to have occurred in the SCS margins solely on the basis of interpretation of seismic data (e.g. [9,10]). Indeed, the seismic transects along the SCS margin show many similarities to the hyperextended Iberian margin, suggesting the possibility of serpentinized mantle in the COT. IODP Expeditions 367/368 were specifically designed to test whether the SCS rifting style and history are comparable to the Iberian margin. The most crucial were the three sites within the COT, where serpentinized peridotite from below the acoustic basement was expected if a model of mantle exhumation similar to that of the Iberian margin is applicable (Fig. 2B-B’) [1].

Serpentinized peridotite, however, was encountered at none of the SCS drill sites (Fig. 3); its absence in all expeditions cast important doubts on models of mantle exhumation [6,7]. Furthermore, the basaltic activity of the late Eocene-early Oligocene (~34–30 Ma) at IODP Sites U1502 in the COT, as evidenced by pillow lava and hydrothermal alternation of both basalt and overlying deep-water sediments, shows a very early initiation of magmatism [7]. Therefore, the IODP drilling indicates a fairly short (<10-myr) rifting phase in the northern SCS, with a rapid transition from rifting to igneous crustal accretion. As such, the northern SCS margin shows ‘marked contrast with the magma-poor Iberia–Newfoundland margins recording more than ~30 Myr of crustal rifting and extensive (> 100 km) subcontinental mantle exhumation prior to igneous crustal accretion’ [11].

According to the Atlantic models, the margin is rifted in one of two ways, depending on the relative timing of rifting and volcanism: either magmatism predates major rift formation (i.e. volcanic type) or rifts form first with prolonged tectonic extension and mantle exhumation (i.e. non-volcanic type) to be followed by magmatism. Obviously, the SCS case supports neither of these two end-member types. The two end-member types do not exhaust the diversity of the Atlantic margin. It has proposed that ‘transitional’ types might exist between the two end-member models; for example, an additional type called the ‘transform continental margin’ has been proposed [12] with the Equatorial Atlantic as an example (ODP Leg 159 [13]). In the following, we argue that the SCS, in the context of converging margins of the Pacific, belongs to a different type of rifted margin from the spectrum of the Atlantic margin. As very few sedimentary basins in the Pacific form in passive margins like those of the Atlantic, we first examine why and how the SCS was identified as an Atlantic-type basin in the past.

## FROM BACK-ARC TO ‘ATLANTIC-TYPE’ BASIN

Suess (1885) was probably the first academic to recognize ‘Pacific and Atlantic types’ of continental margins, later labeled as active and passive margins [14]. Historically, our knowledge of passive margins is largely tied to the discoveries of giant oil-bearing sedimentary basins. Ocean scientific drilling has always recognized two distinct continental margins with different scientific foci: sedimentary basins in the Atlantic passive margin and earthquakes and island arcs in the Pacific active margin.

Marginal basins in the Western Pacific came into sight of the scientific community along with sea-floor spreading. Karig [15] first defined the marginal basins as semi-isolated basins or series of basins behind the volcanic chains of island arcs. He compared the marginal basins with ‘small ocean basins’ of Menard [16] and ascribed their origin to crustal extension. To resolve the paradox of how extension can occur in the compressive plate boundaries of the Pacific margins, a back-arc model was proposed where the tensional forces are caused by oceanic trench rollback [15]. At this stage, the SCS was considered as such a back-arc basin opened as an ‘inter-arc basin’ behind the Philippine arc system [17]. However, the back-arc model of SCS opening was disproved by the subsequent identification of magnetic lineations. The magnetic anomaly data acquired in 1979 showed east-trending lineations in the eastern sub-basin of the SCS, which helped dating the age of the SCS sea-floor spreading to the late Oligocene to early Miocene. The direction and age of these magnetic lineations are incompatible with those of the Philippine arc and thus invalidate the back-arc model of SCS formation [18,19].

The SCS measures 3.5 million km^2^ in area—much smaller than that of the Atlantic (100 million km^2^). Regardless of the size differences, however, the SCS basin does show some similarities to the Atlantic. Because the symmetric pattern of the east-trending magnetic lineations and the COT development are in some way similar to those in the Atlantic, the SCS was considered as an ‘Atlantic-type’ marginal basin, bounded by passive continental margins to the north and south [19]. This interpretation coincided with the rapid development of oil exploration in the SCS in the 1980s, when a close tie between oil reservoirs and passive margins greatly enhanced the significance of the SCS for the research community.

Over several decades, passive continental margins have remained the exploration frontiers for the oil industry. The post-rift sequences of passive margins are estimated to host approximately 35% of all giant field discoveries, which in turn represent 67% of discovered conventional hydrocarbons [20]. This explains the enthusiasm in searching for passive margin basins, in particular non-volcanic rifted basins, in the global ocean and continents. At the same time, passive margins have always been at the core of scientific planning of the 50 years of international ocean drilling. Remarkable examples of planning activities include the IPOD Passive Margin Advisory Panel in the 1970s [21] and the Continental Breakup and Birth of Oceans Mission (COBBOOM) in the 2000s [22]. The Iberian model of the non-volcanic passive margin has been applied to paleo- and modern basins, from the Tethys Ocean in the Alps to the SCS in the Pacific [23].

**Figure 4:**
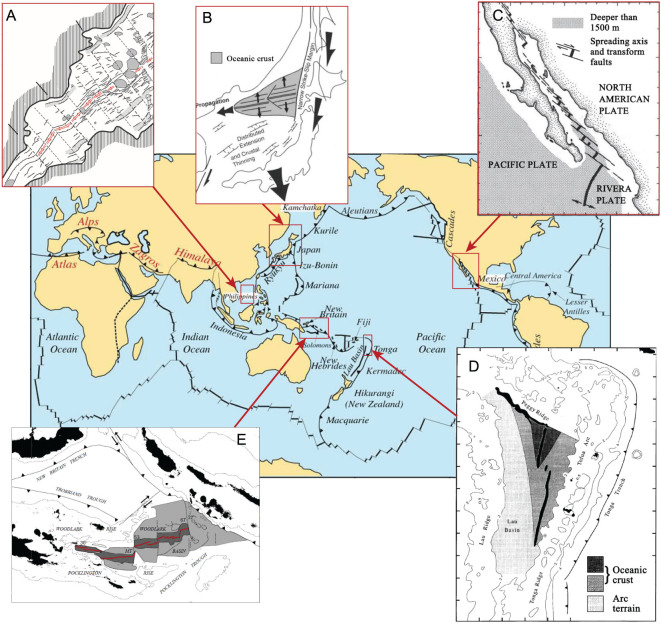
Rift propagation in plate-edge basins. (A) South China Sea, SW sub-basin [33]; (B) Sea of Japan [34]; (C) Gulf of California [35]; (D) Lau Basin [36]; (E) Woodlark Basin [37].

In the recent literature, the SCS is widely cited as a typical example of magma-poor rifted margins (e.g. [24]). Using the Iberian model as a template for interpretation of seismic data, ‘serpentinized mantle’ and ‘zone of exhumed continental mantle (ZECM)’ have been proposed for deep structures along many of the SCS seismic transects (e.g. [9,10,25], to name a few). Noteworthy is the northern margin transect interpreted as a ‘zone of exhumed continental mantle’ by Franke *et al.* (Fig. 10 of Ref. [10]), which almost overlaps the transect of the SCS IODP drilling (Fig. 2B-B’); however, recent drilling did not support this interpretation of exhumed mantle. Therefore, time is ripe to reexamine not only all the seismic data previously used to interpret serpentinized mantle, but also the universality of the current concept of two types of passive margins.

## PLATE-EDGE *VS* INTRA-PLATE RIFTING

The concept of volcanic *vs* non-volcanic end-members is convenient in application, as the two types are readily recognizable in images of seismic transects. This simple binary dichotomy itself, however, did not offer understanding of the fundamental processes causing the observed variations in the rifting continuum. Nearly two decades ago, Wilson *et al.* [23] realized that ‘it may be premature to use models based on the Iberia and Tethyan margins as the paradigm for all non-volcanic margins’. This particularly applies to basins outside the Atlantic. For example, the Woodlark Basin and Gulf of California in the Pacific are active rifting systems with transition from rifting to sea-floor spreading, but neither can be assigned to the volcanic/non-volcanic grouping [22].

The SCS margin does not show any traces of SDRs and thus would be considered a ‘non-volcanic’ margin. However, the recent ocean drilling revealed extensive volcanic activities in the Eocene, during the Oligocene-early Miocene drifting, and in periods after sea-floor spreading [26], as evidenced by recovered multiple seamount valcaniclastic layers in the SCS sub-basins [27]. Also remarkable is the recent discovery of primary carbonates in the Miocene volcanic clasts near the relict-spreading ridge in the SCS eastern sub-basin, which are interpreted to have originated from carbonated silicate melts through recycling of subducted oceanic crust [28]. The large volume of post-spreading volcanism in the SCS, in the forms of seamounts, intrusions and underplating, can be explained by hypothetical subduction-induced mantle flows, which is supported by recent geophysical observations and modeling [29].

Of particular importance is not only the volume of magmatism, but also its timing: the Eocene magmatism before rifting and spreading should negate the ‘non-volcanic’ label for the SCS basin. Thus, the SCS belongs neither to a ‘non-volcanic’ nor ‘volcanic’ group. Furthermore, evidence is mounting that the SCS is also not a margin structure between the two end-members. As discussed below, the fundamental differences probably lie in the deeper lithosphere below the Atlantic and Pacific margins, and hence the Atlantic-based passive margin classification cannot be applied to the Pacific.

For the North Atlantic, the Iberian Peninsula today is formed from Hercynian Massif made up of deformed and metamorphosed Precambrian and Paleozoic rocks intruded by large granitoid batholiths during and after the Hercynian continent–continent collision. The long history of the Iberian Basin opening is predated by a pre-rift phase with crustal thickening and magmatic additions taking place in a convergent margin setting [30]. This is in a sharp contrast to the Western Pacific margin, including the SCS, where the lithosphere might be significantly weaker. As a super-ocean margin, the Western Pacific has been a region of long-lived subduction since perhaps 450 Ma and a total length of about 30 000 km of lithosphere slabs have likely subducted here during the last 150 Ma, turning the region into a ‘slab graveyard’. The subduction also brought a large amount of water into the mantle, which may have lowered the solidus temperature and viscosity of the mantle peridotite. Consequently, lithospheric break-up in this region might be much easier than in the Atlantic [31,32].

**Table 1:** Two genetic types of rifted basins.

Rifting type	Intra-plate	Plate-edge
Position	Inside continental plate	Near subduction zone
Produced basin	Ocean	Marginal sea
Example	Atlantic	South China Sea
Stage in Wilson cycle	Supercontinent collapsing	After collapsing
Possibly involved mantle cycle	Entire mantle	Upper mantle
Duration of opening process	10^8^ years	10^6^–10^7^ years
Geographic size	10^7^ km	10^5^–10^6^ km

The different nature of lithosphere between the Atlantic and Pacific settings most likely accounts for their differences in the rifting process and rift-to-drift transition. Therefore, we propose to distinguish two types of rifted basins: an intra-plate type exemplified by Atlantic basins and a plate-edge type including the SCS (Table 1). Despite some superficial resemblance, the intra-plate and plate-edge types represent two radically different processes of rifting and subsequent transition to sea-floor spreading. For the intra-plate type, the break-up of the continental lithosphere lasted for a very long period of time, such as from the late Triassic to early Cretaceous for the Iberian margin; but, for the plate-edge type, as seen from the SCS, rifting and transition to sea-floor spreading are much more rapid. As summarized in Table 1 and discussed below, the two types of rifted basins belong to two different stages in the Wilson cycle and they differ not only in the rifting and spreading opening processes and speed, basin lifespan and geographic size, but probably also in mantle cycling underneath.

The proposed plate-edge type of rifted margin is not new to the tectonic research community. The Woodlark Basin, Lau Bain and Gulf of California in the Pacific (Fig. 4) are all active rifting basins and their origins remain a subject of debate. Like the SCS, we argue that these are plate-edge rifting basins different from the intra-plate rifting type in the Atlantic and they lack the characteristics of the volcanic or non-volcanic end-members. To compare the opening process of the SCS basin with those from the Atlantic without solid drilling evidence is likely to be misleading. These plate-edge rift basins show significant similarity in their genesis and evolution that should no longer be ignored.

The plate-edge rifted basins are typically not as long-lived as large-scale ocean basins because the opening and closing of these basins are often related to subduction dynamics. The Woodlark Basin (Fig. 4E), for example, is a typical back-arc basin, formed in a complex way from subduction of the Pacific Plate under the Australian Plate. Its sea-floor spreading initiated in the eastern Woodlark Basin before ~6 Ma and propagated westward in a step-wise, discontinuous fashion at an average propagation rate of 14 cm/yr [38]. Another frequently cited example of passive margins in the Pacific is the Gulf of California, which formed from subduction of the Farallon Plate under North America and evolved during extension between Baja California and mainland Mexico [39]. Noticeable is the rapid rupture of the lithosphere, as sea-floor spreading commenced in the southern Gulf of California only ~6–10 Ma after the formation of the oblique-divergent plate boundary at ~12.5 Ma; this is in contrast to 30–80 Ma for rift development in the interior of continents before the onset of sea-floor spreading [40] (Table 1).

For the intra-plate margins in the Atlantic, extension was both slow (<2 cm/yr) and prolonged, contrasting greatly with the plate-edge basins. For example, the Woodlark Basin opens much more quickly (3–7 cm/yr), resulting in a very narrow COT and a sharp boundary between continental rifting and sea-floor spreading. This is in stark contrast to the break-up of the Newfoundland and Iberia conjugate margins, which are associated with ultra-slow spreading in the early North Atlantic and extremely wide transition zones of up to 150 km [41]. The narrow COT has been noticed as another distinct feature for the SCS [42], the Gulf of California, as well as in the Bande Sea [43,44] and might be one of common characteristics of the plate-edge rifting basins.

Another possible common feature is the age–depth relationship. It has long been known that the marginal basins in the Western Pacific are deeper than expected based on the standard age–depth curves from the major oceans [45]. The basement depth of the Philippine Sea is about 800 m deeper than that of the major ocean floors of the same age [46], whereas, in the Banda Sea, the basement-depth anomaly might be as much as 2000 m [47]. It remains unclear what is the cause of the difference, but its effect is recorded in the paleo-bathymetry records of the basin evolution. In the SCS, for example, deep-water conditions prevailed in the northern margin already at the beginning of sea-floor spreading [48].

**Figure 5:**
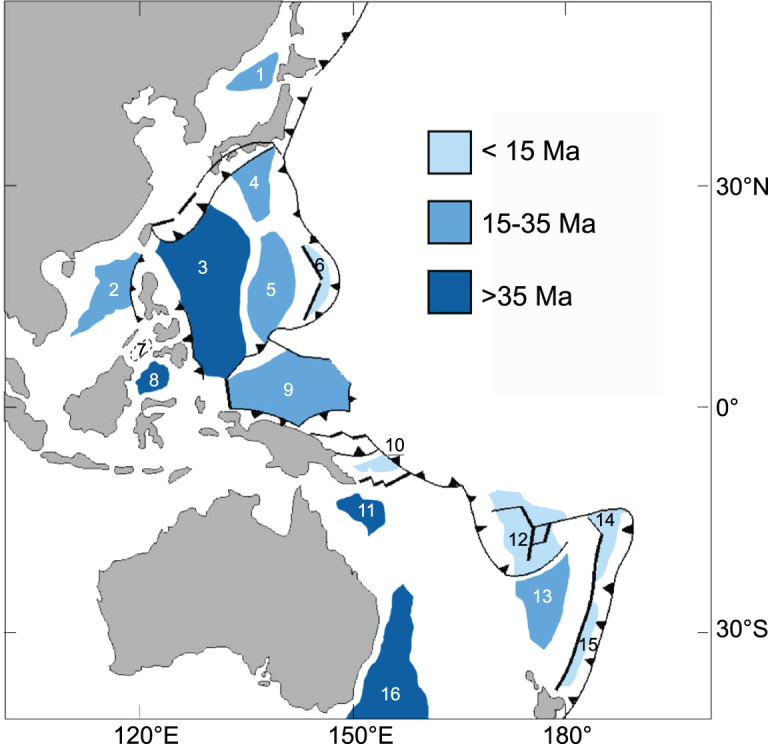
Age distribution of ocean crust in major West Pacific marginal basins (numbers as listed in Table 2).

In recent years, the fundamental differences between plate-edge rifting from intra-plate rifting have been highlighted (such as [49]), although they have not yet attracted sufficient attention from the broad research community. For example, looking into the cause of rapid rupture of continental lithosphere in the Gulf of California, Umhoefer [40] pointed to its location at a tectonically active margin since the Jurassic. In a comparison of Iberia–Newfoundland, Central South Atlantic and SCS basins, Brune *et al.* [25] distinguished two ways of rifting: intra-continental rifting and back-arc rifting. It was proposed that the intra-continental rifting may lead to separation of major landmasses, thereby generating a new ocean basin; on the other hand, ‘marginal rifts often form as back-arc basins in response to subduction dynamics’ [25]. Clearly, the above studies have articulated that the distinction of plate-edge rifting from intra-plate rifting is needed for fully understanding the rifting processes of marginal basins.

## WESTERN PACIFIC SYSTEM OF MARGINAL BASINS

In the modern world, the plate-edge rifted basins are concentrated in the Western Pacific, which includes more than 75% of the global marginal basins [50]. Karig [13] was probably the first to consider the Western Pacific marginal basins as an interconnected system and proposed their back-arc (‘inter-arc’) origin. However, this simplistic view about their formation was challenged by the subsequent discoveries of the highly diverse structure and history of the Western Pacific marginal basins. In a more systematic discussion of basin origin, Tamaki and Honza [50] distinguished back-arc basins from other marginal-basin types, such as basins with trapped oceanic crust or basins unrelated to subduction. Of particular interest are the common characteristics of the marginal basins summarized by these studies, including their relatively short lifespan resulting from their destruction after the cessation of spreading, their relatively young age (<80 Ma) in the context of the long history of plate subduction (>180 Ma) and the changing trend of spreading axes due to the effects of the surrounding tectonic settings.

We believe that the Western Pacific marginal seas are interconnected in their formation regardless of their diversity in shape and size. As indicated in Fig. 5 and Table 2, the oceanic crust of Western Pacific marginal seas mostly formed in the middle and late Cenozoic and their crust ages, in general, become younger from west to east, corresponding well to the model of back-arc extension with eastward rollback of the subducting plate [51]. Furthermore, recent studies have provided a strong hint that reorganization of the Pacific Plate around 50 Ma kicked off the formation process of the marginal-basin system [52,53], but it was also the time of India–Eurasia collision [54]. Convergence of the three plates (Eurasian, Pacific, Indian–Australian) have caused great complexity in the formation history of marginal seas. Attempts have been made to reconstruct marginal-basin development in the northwestern and southwestern Pacific [55–57], revealing the possible genetic link between the basins, as well as a complex history of destruction and reorganization after basin formation. Although the data from the deep mantle are still extremely limited for now, these attempts offer new approaches to explore the inter-basin relationship and formation of individual margin basins. This applies particularly well to the Philippine Sea basin. After a history of long-distance travel and rotation, it is extremely difficult for the evolution of the West Philippine Sea to be properly recognized from the modern geography [58,59].

**Table 2:** Age of oceanic crust in major marginal basins of Western Pacific (see Fig. 5).

No.	Basin	Age (Ma)	References
1	Sea of Japan	24–18	Tamaki *et al.*, 1992 [34]
2	South China Sea	34–15	Li *et al.*, 2015 [27]
3	West Philippine Sea	55–33	Honza and Fujioka, 2004 [60]
4	Shikoku	25–15	
5	Parece Vela	29–15	
6	Mariana	15–0	
7	Sulu Sea	15–10^*^	Silver *et al.*, 1991 [61]
8	Celebes Sea	45–35	
9	Caroline	35–15	Dong *et al.*, 2017 [62]
10	Woodlark	6–0	Taylor *et al.*, 1995 [63]
11	Coral Sea	~65–52	Schellert *et al.*, 2006 [56]
12	North Fiji	12–0	
13	South Fuji	35–24	
14	Lau	6–0	
15	Havre	6–0	
16	Tasmania Sea	80–52	

^*^The nature of ‘oceanic crust’ in the Sulu Sea was questioned and the recovered basalt by ODP drilling might be from a subsided volcanic arc [64].

The origin of plate-edge basins is often associated with plate subduction, but the great variety in their formation cannot be convincingly demonstrated by the simple 2D concept of back-arc rifting [51]. Recent seismic anisotropy studies have revealed 3D mantle flow caused by subducting slabs [65], which can also be considered as a force contributing to plate-edge rifting. In fact, the multiple types of rifting basins were realized much earlier. For example, in their review on the Western Pacific marginal basins, Jolivet *et al.* [66] distinguished two ways of opening: trench suction and intercontinental deformation, with the Mariana Trench and the SCS as respective examples. In view of plate tectonics, the trench suction type is driven by convergence of two oceanic plates, whereas the intercontinental deformation type is by convergence between oceanic and continental plates. The Mariana Trough opened as a typical back-arc rift, but the formation of the SCS could be related to oblique right-slip shear in East Asia: the motion of the strike-slip faults led to the development of rectangular-shaped back-arc basins including not only the SCS, but also the Sea of Japan and Sea of Okhotsk [67,68].

**Figure 6:**
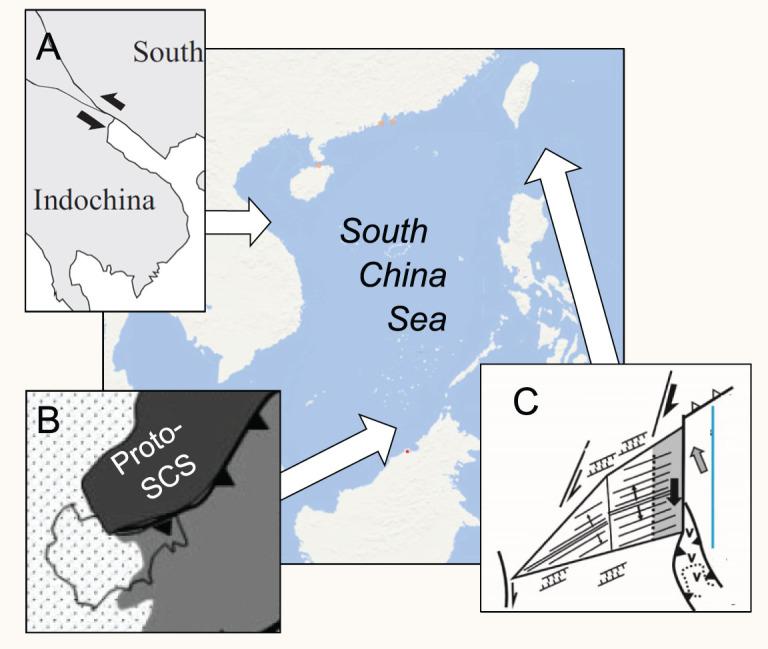
Tectonic models for the SCS formation. (A) Extrusion model (forcing from the west); (B) Proto-SCS model (forcing from the south); (C) strike-slip faulting model (forcing from the east).

Since the SCS is a member of the system of marginal basins in the Western Pacific, its formation should no longer be studied in isolation. The evolution of the SCS has always been closely interconnected with its neighboring basins, particularly the West Philippine Sea. This point deserves special attention because a large part of the SCS in the east has already disappeared since the end of the SCS sea-floor spreading due to its subduction under the Philippine Sea [69]. The discovery of Eocene deep-sea sediments and Eocene basalts during recent IODP Expeditions [70] provides evidence to support the idea that the opening history of the SCS was closely related to that of the West Philippine Sea [69].

After the recognition of the magnetic anomalies in the SCS basin, various models have been proposed to explain its opening. There are two contrasting end-members: the collision–extrusion model (Fig. 6A) attributes the SCS opining to the SE displacement of the Indochina Block driven by India–Asia collision (e.g. [71,72]), while the subduction–collision model (Fig. 6B) suggests the SCS opened in response to slab pull during subduction of proto-South China Sea oceanic crust (e.g. [73–75]). If the modern SCS is a relict of a much larger basin and if the SCS history is strongly linked to the Philippine Sea Plate evolution, it would be logical to look for its origin on its eastern border in connection with the West Philippine Sea basin. We suggest, therefore, that the SCS was separated from the Eurasian continent along strike-slip faults inherited from Late Mesozoic, followed by lithospheric stretching along the Eurasian/Huatung Plate boundary in the Early Cenozoic (Fig. 6C; [68]). We also suggest that, while the strike-slip faults on the west (Fig. 6A) and subduction in the south (Fig. 6B) must also play their roles in the process, the key force responsible for the opening to the east of the SCS should not be overlooked (Fig. 6C).

**Figure 7:**
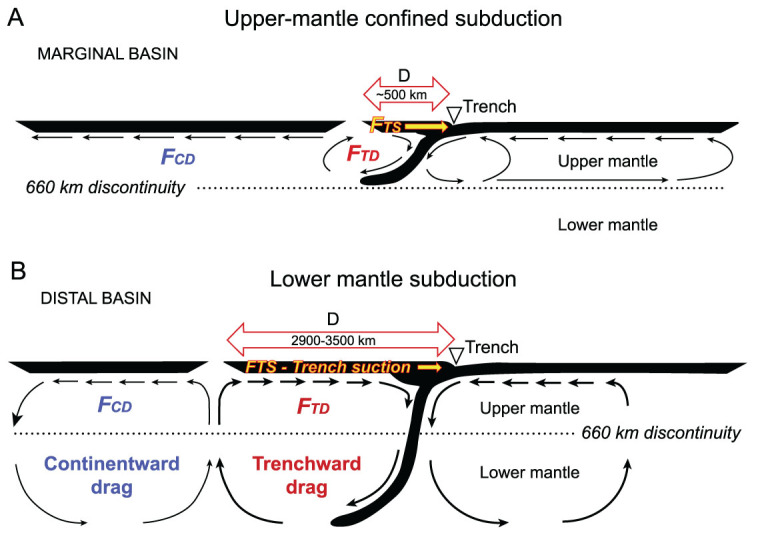
Two types of mantle circulation involved in the formation of (A) plate-edge *vs* (B) intra-plate rifted basins (modified from [83]).

## RIFT BASINS IN THE WILSON CYCLE

Our knowledge of ocean-basin geodynamics has been greatly advanced over the past 50 years, but a spatial imbalance in data sources may lead to some biases in interpretation and modeling. Compared to the Atlantic, the Western Pacific remains much less well studied, partially due to the complexity of regional geology. The Atlantic is a region of plate divergence and its geological record is largely preserved, whereas, in the Western Pacific, a large portion of its records have disappeared by subduction. The Western Pacific marginal basins comprise the marine part of the so-called ‘Western Pacific Triangular Zone’, where the hydrous mantle is most dynamic in modern Earth [32]. It has been argued that the driving forces of tectonic processes in the West Pacific are deeply rooted in the mantle.

Fifty years ago, the continental margin basins were studied as ‘modern geosynclines’ based on the thick accumulation of sediments along the margins [76]. Afterwards, various tectonic concepts of passive margins were developed with sedimentary basins as the research focus. As most of the world's rifted margins have now been imaged by seismic reflection and new technologies allow ever-deeper penetration and better resolution, the influx of new data challenges the tectonic concepts developed solely on the basis of the upper layers of the lithosphere [77]. In the case of SCS, recent contributions from tomography start to unveil the mystery of subducted slabs and shed new light on its opening (e.g. [74,78,79]).

When the two types of rifting are examined in the framework of the Wilson cycle, two different stages of development can be recognized. Intra-plate rifting occurred in the Mesozoic, giving rise to the major ocean basins; and plate-edge rifting occurred mostly in the middle and late Cenozoic, leading to the formation of marginal basins (Fig. 5 and Table 2). In the Wilson cycle, intra-plate rifting coincided with the break-up of the Pangea supercontinent, whereas plate-edge rifting occurred at a later stage in the cycle, during the subducting stage [80], roughly corresponding to the ‘two prominent periods of enhanced rifting’ at 160–100 Ma and after 66 Ma, respectively [81]. These analyses explain the difference of the two rifting types in their development stages and lifespan. According to the statistics over 2740 Ma of geological history, the ancient passive margins have a mean lifespan of 181 Ma [82], indicating much longer development of intra-plate rifted basins, which is in contrast with the much shorter lifespan of 10^0^–10^1^ Ma for the plate-edge counterparts (Table 1).

The profound contrast between the two types of rifting becomes even more obvious when the basin development is examined together with mantle flow in the deep Earth. As shown by numerical modeling, the contribution of subduction and the coupling of mantle flow with rifting and drifting continents depend on the depth of subduction. If subduction is confined only to the upper mantle, rifting might be expected to occur at the plate edge (Fig. 7A). In contrast, if subduction slabs reach deep into the lower mantle, the entire mantle flow is expected, leading to collapsing of supercontinents with intra-plate rifting (Fig. 7B) [83]. In a global context, the two rifting stages discussed above may ultimately be related to the ‘geotectonic bipolarity’ at the mantle base, which is responsible for the break-up of Pangea and the birth of the Pacific Plate [84]. Clearly, the driving forces and causal mechanism of the rifting–drifting processes are deeply rooted in the mantle, beyond the access of classical geological–geophysical approaches. The target of future investigations of the rifted basins in the Western Pacific must focus more on deep processes to improve our understanding of the ‘Earth connection’—the connection of the surface processes with those in the deep Earth [85].

## CONCLUSIONS

The recent drilling results of IODP Expeditions 367/368/368X challenge the applicability of the Iberian model of the non-volcanic passive margin to the SCS. Specifically, the absence of serpentinized peridotite at the COT sites disproved the pre-cruise interpreted mantle exhumation; meanwhile, the recovery of the MORB-type Eocene basalt implies a rapid rift-to-igneous crustal-accretion transition. The differences in rifting and rift-to-drift transform indicate two types of continental lithosphere rifting processes: intra-plate type for the Atlantic and plate-edge type for the SCS. The concept of two end-members of continent rifting (i.e. volcanic and non-volcanic) in the Atlantic does not apply to the plate-edge rifted basins in the Pacific.

The two types of continent rifting occur at two different stages in the Wilson cycle. Intra-plate rifting occurs at an early stage in the Wilson cycle and is associated with continental break-up, whereas the plate-edge rifting takes place much later. Thus, the two types of rifted basins differ from each other not only in structure and formation process, but also in their lifespan and geographic size (Table 1). As currently understood, plate-edge basins comprise a separate type of rifting from the classic intra-rifting and we call for an end to their consideration as an exception to the Atlantic stereotype.

In the modern world, three-quarters of the plate-edge rifted basins are concentrated in the Western Pacific, along the largest subduction zone of the global ocean. The Western Pacific marginal basins constitute a system where the individual basins are interconnected in their formation and evolution. Consequently, the development of the SCS should no longer be studied in isolation, but in the context of the entire system to include all its neighboring basins, as well as be compared to modern active plate-edge basins such as the Woodlark Basin and the Gulf of California.

Unlike the intra-plate rifted basins, the plate-edge basins have their driving forces and causal mechanism of evolution related to subducting slabs deep in the mantle, beyond the access of classical geological and shallow geophysical approaches. A new generation of geodynamic studies is required to unveil the mechanism of the Western Pacific system of marginal basins. This new challenge calls for further development of deep-sea drilling into the basement rocks in rifted basins and technical improvement of geophysical explorations of the deep lithosphere.
